# The benefits and tolerance of exercise in myasthenia gravis (MGEX): study protocol for a randomised controlled trial

**DOI:** 10.1186/s13063-017-2433-2

**Published:** 2018-01-18

**Authors:** Simone Birnbaum, Jean-Yves Hogrel, Raphael Porcher, Pierre Portero, Bernard Clair, Bruno Eymard, Sophie Demeret, Guillaume Bassez, Marcela Gargiulo, Estelle Louët, Sonia Berrih-Aknin, Asmaa Jobic, Philippe Aegerter, Philippe Thoumie, Tarek Sharshar, the MGEX Study Group, Philippe Aegerter, Guillaume Bassez, Sonia Berrih-Aknin, Anthony Behin, Simone Birnbaum, Francis Bolgert, Bernard Clair, Sophie Demeret, Nawal Derridj Ait-Younes, Nabila-Yasmine Domingo, Bruno Eymard, Diane Friedman, Melinee Frenkian, Marcela Gargiulo, Jean-Yves Hogrel, Asmaa Jobic, Pascal Laforet, Isabelle Ledoux, Estelle Louet, Judith Mendelson, Sandra Misdrahi, Cecilia Orblin Bedos, Raphael Porcher, Pierre Portero, Benjamin Rohaut, Tarek Sharshar, Elodie Soler, Philippe Thoumie, Frederique Truffault, Nicolas Weiss, Linda William

**Affiliations:** 1 Institute of Myology, Pitié-Salpêtrière Hospital, Assistance Publique–Hôpitaux de Paris (AP-HP), Paris, France; 2 Bioingénierie, Tissus et Neuroplasticité (BIOTN) EA 7377, University Paris-Est, UPEC, Créteil, France; 3 Centre de Recherche Épidémiologie et Statistique Sorbonne Paris Cité (CRESS-UMR1153), Hôtel-Dieu, AP-HP, Paris, France; 4 Rothschild Hospital, AP-HP, Paris, France; 5 Intensive Care Unit, Raymond Poincaré Hospital, AP-HP, Garches, France; 6 Neurological Intensive Care Unit, Pitié-Salpêtrière Hospital, Paris, France; 7 Laboratory of Clinical Psychology, Psychopathology, and Psychoanalysis (EA 4056) Paris Descartes University – Sorbonne Paris Cité, Paris, France; 8 UMRS 974 UPMC, INSERM, FRE 3617 CNRS, AIM, Centre of Research in Myology, Paris, France; 9 Unité de Recherche Clinique Paris ÎIle- de- France Ouest (URCPO), Ambroise Paré Hospital, Boulogne Billancourt, France; 10 Raymond Poincaré Hospital, AP-HP, Garches, France; 11 UVSQ, UMR-S 1168, Université Versailles St-Quentin-en-Yvelines, Versailles, France; 12 INSERM, U1168 VIMA, Villejuif, France; 13 University of Versailles, Saint-Quentin-en-Yvelines, France; 14 Department of Histopathology and Animal Models, Institut Pasteur, Paris, France

**Keywords:** Myasthenia gravis, Physical exercise, Quality of life

## Abstract

**Background:**

Research exploring the effects of physical exercise in auto-immune myasthenia gravis (MG) is scarce. The few existing studies present methodological shortcomings limiting the conclusions and generalisability of results. It is hypothesised that exercise could have positive physical, psychological as well as immunomodulatory effects and may be a beneficial addition to current pharmacological management of this chronic disease. The aim of this study is to evaluate the benefits on perceived quality of life (QOL) and physical fitness of a home-based physical exercise program compared to usual care, for patients with stabilised, generalised auto-immune MG.

**Methods:**

MGEX is a multi-centre, interventional, randomised, single-blind, two-arm parallel group, controlled trial. Forty-two patients will be recruited, aged 18–70 years. Following a three-month observation period, patients will be randomised into a control or experimental group. The experimental group will undertake a 40-min home-based physical exercise program using a rowing machine, three times a week for three months, as an add-on to usual care. The control group will receive usual care with no additional treatment. All patients will be followed up for a further three months. The primary outcome is the mean change in *MGQOL-15-F* score between three and six months (i.e. pre-intervention and immediately post-intervention periods). The *MGQOL-15-F* is an MG-specific patient-reported QOL questionnaire. Secondary outcomes include the evaluation of deficits and functional limitations via MG-specific clinical scores (Myasthenia Muscle Score and MG-Activities of Daily Living scale), muscle force and fatigue, respiratory function, free-living physical activity as well as evaluations of anxiety, depression, self-esteem and overall QOL with the WHO-QOL BREF questionnaire. Exercise workload will be assessed as well as multiple safety measures (ECG, biological markers, medication type and dosage and any disease exacerbation or crisis).

**Discussion:**

This is the largest randomised controlled trial to date evaluating the benefits and tolerance of physical exercise in this patient population. The comprehensive evaluations using standardised outcome measures should provide much awaited information for both patients and the scientific community. This study is ongoing.

**Trial registration:**

ClinicalTrials.gov, NCT02066519. Registered on 13 January 2014.

**Electronic supplementary material:**

The online version of this article (10.1186/s13063-017-2433-2) contains supplementary material, which is available to authorized users.

## Background

Auto-immune myasthenia gravis (MG) is a rare, chronic, auto-immune disease of the neuromuscular junction. The incidence is estimated to be 30 per 1,000,000 people per year worldwide [1]. MG affects both genders and symptoms include fluctuating fatigue and weakness which vary in intensity and location (upper and/or lower limbs, axial, eyes and face, bulbar and respiratory muscles) [2]. These symptoms affect patients to varying degrees and while disease-induced mortality is rare nowadays, activities of daily function are affected and quality of life (QOL) is reduced [3–6]. Relapses are unpredictable and can be present throughout the disease course, especially in the initial stages when the disease may not yet be treated. Current treatment includes various pharmacological agents which are either symptomatic such as acetylcholinesterase inhibitors which act to temporarily relieve symptoms or disease-modifying immunosuppressors aimed at minimising the auto-immune response. As there is currently no known cure, the goal of treatment is to obtain remission; and where this is not possible, to obtain disease stability with the least symptoms.

While rest is recommended during an acute exacerbation of MG, evidence is lacking as to whether exercise is both feasible and beneficial in periods of disease stability. Anecdotal evidence from patients with MG suggests that those who participate in regular physical exercise feel better. According to them, it gives them confidence in their capacities and helps them understand their disease and their personal limits. Some even report experiencing symptom improvement and reduced levels of fatigue [7].

For the general population, it is widely accepted that regular physical exercise has multiple positive physical and psychological effects as well as being effective in the prevention of various non-communicable diseases such as stroke, heart disease and cancer and increasing longevity [8–10]. Recently, the use of exercise has been proposed in the management of various neurological diseases [11–13]. Exercise has even been compared to pharmacological treatment and has been found to be equivalent in some populations, supporting the recent notion that ‘exercise is medicine’ [14]. Traditionally, exercise was thought to exacerbate symptoms for patients suffering from MG as their muscle weakness and fatigue was thought to be brought on or worsened by physical exertion and improved with rest. Despite the increasing number of studies exploring exercise in various neuromuscular pathologies, to date, little research has explored physical activity in the context of MG [15, 16]. Several case studies exist which demonstrate that despite the disease patients can participate in sport and increase their strength, power or endurance with adequate training [17–20]. A within-subject control study provided promising results, yet these limited data do not enable us to draw reliable conclusions concerning the safety and benefits of physical exercise for patients with MG [21]. Two new studies provide further information including different types of physical exercise [22, 23]. In the study by Rahbek et al., progressive resistance training appears to be beneficial however aerobic training seems to increase depression and fatigue and reduce knee extensor strength [22]. The mixed aerobic resistance program study carried out by Westerberg improved functional physical performance and exercise self-efficacy without strength improvements noted [23]. Exercise was well tolerated in both studies; however, due to small sample sizes and lack of control group, it is difficult to make firm conclusions about the various possible benefits. It is hypothesised that physical exercise is beneficial for adults with stabilised MG.

The primary aim of this study is to determine whether a regular moderate intensity physical exercise program improves perceived QOL for patients with stable, generalised MG. Secondary aims are to evaluate the effects of this exercise program compared to usual care on physical fitness, depression and self-esteem as well as on the immune system.

## Methods/Design

### Study design, ethics, consent and permission

**Fig 1:**
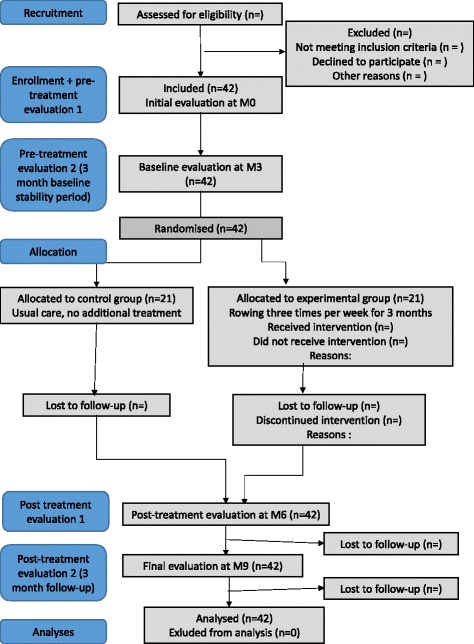
CONSORT *flow diagram* of the MGEX study

**Fig 2:**
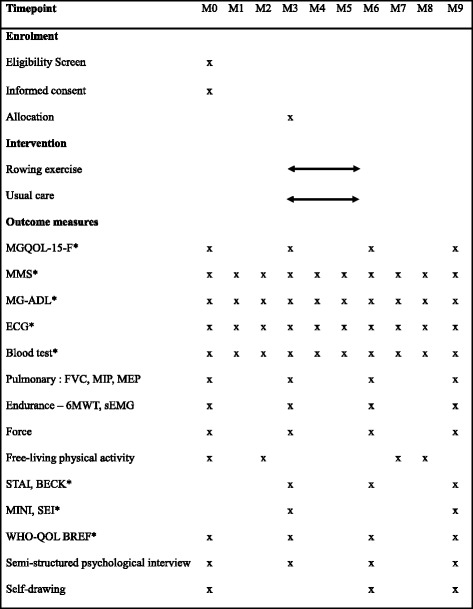
Standard protocol items recommendations for interventional trials (SPIRIT). Schedule of enrolment, intervention and assessments. M0 includes enrolment and informed consent signing. Visits are monthly (M). Randomisation and allocation takes place following the 3-month visit (M3). Intervention period is 3–6 months. Post-intervention evaluation is at M6. *Evaluator blinded to treatment allocation

This is a multi-centre, interventional, randomised, single-blind, parallel group, controlled trial with a planned duration of four years (nine months for each patient) (Figs. 1 and 2).

Safety evaluations are monthly; muscular and psychological evaluations are three-monthly (M0, M3, M6 and M9). The intervention period is between M3 and M6. The study has been approved by an ethics committee in accordance with French regulations (*Comité de Protection des Personnes Ile de France XI* under the CPP number #13064 on 13 December 2013 and authorised by the *Agence National de Sécurité du Médicament et des produits de santé* on 11 October 2013). The trial is conducted in accordance with the Helsinki Declaration [24]. Each participating patient receives written and verbal explanations about the study and evaluation procedures. The trial is registered in the ClinicalTrials.gov registry (NCT02066519). Reporting of the study will adhere to the Consolidated Standards of Reporting Trials (CONSORT) guidelines and the Standard Protocol Items: Recommendations for Interventional Trials (SPIRIT) [25–27]. The SPIRIT checklist is available as an Additional file 1.

Participating centres include the neuromuscular and neurological intensive care departments, Pitié-Salpêtrière Hospital, Paris, the Raymond Poincaré Hospital, Garches (Paris region) and Rothschild Hospital, Paris.

### Sample size calculation

We estimated that 42 patients (21 in each arm with 1:1 randomisation) would be necessary to show a difference in changes in the *MGQOL-15-F* of 8 points between both arms with 80% power and a two-sided type I error rate of 5%, assuming the standard deviation of changes in the *MQGOL-15-F* is 9 points based on Burns et al. [28]. A difference in 8 points is considered to be a clinically meaningful change for this population. No increase in sample size for potential drop-outs was considered in the calculation.

### Recruitment and trial participants

All patients presenting for a regular out-patient consultation with their neurologist in one of two university hospitals in Île-de-France, France (Pitié-Salpêtrière, Paris and Raymond Poincaré, Garches), are screened for eligibility. Both hospitals are specialist centres for the management of MG. Eligible patients are invited to participate and a date is organised for study inclusion. Information about the study is also advertised by the French Association against Neuromuscular Disorders (AFM) myasthenia group and various social media patient groups. Information flyers are displayed in the two university hospital waiting rooms.

Adult patients with a diagnosis of mild or moderate generalised MG (II or III on the Myasthenia Gravis Foundation of America [MGFA] classification) are considered for eligibility [29]. Diagnosis should be confirmed by the presence of circulating antibodies against acetylcholine (AChR) or muscle-specific kinase (MuSK) or low density lipoprotein receptor-related protein 4 (LRP4) receptors or abnormal repetitive nerve stimulation testing (decrement > 10%) on EMG or abnormal single fibre EMG (conduction block or jitter) or based on their clinical history and symptom improvement with acetylcholinesterase inhibitors. The MG must be stabilised for at least six months before entry into the study; thus, duration of the disease must be at least six months but is otherwise unrestricted. Both men and women aged 18–70 years are eligible for inclusion. For practical reasons (due to multiple visits and rowing machine delivery), participants should live in Paris or in the surrounding Paris region; they agree to be able to participate in the exercise program and can house the rowing machine for the three-month duration in their home. They should be affiliated with the national social security system.

Exclusion/non-inclusion criteria:

Patients participating in other interventional clinical trials in the preceding three months, patients for whom intensive physical exercise would be contraindicated because of unstable coronary syndrome or myocardial infarction within the preceding three months, respiratory failure (vital capacity < 70% predicted value) or cardiac failure (ejection fraction < 50% predicted value), other neuromuscular pathology, disabling rheumatological disease (> 80% disability on the Barthel scale), chronic pain or disabling orthopaedic conditions, hospitalisation in the last three months for a serious medical or surgical condition, anaemia (haematocrit < 30%), stroke within the previous year. Pregnancy or patients with pure ocular or severe MG (MGFA class I, IV or V) or with severe cognitive impairment necessitating specific protection are not eligible for inclusion, nor are patients with a score < 15 on the *MGQOL-15-F*.

### Randomisation, allocation and blinding

Figure 1 depicts the participants’ flow through the study according to the CONSORT guidelines [25, 26]. Figure 2 presents the timeline of assessments and intervention. Following study inclusion, there is a three-month observation period to ensure disease stability (in addition to the six-month stability required before inclusion). All consecutive participants are then randomly assigned to either the control or experimental group with a 1:1 allocation ratio as per a computer-generated randomisation schema stratified by centre using permuted blocks of randomly varying sizes. To ensure concealment, the block sizes are not disclosed. Group allocation is revealed via a computer software (CleanWeb) exclusively to the non-blinded physiotherapist once randomisation has been performed (concealed allocation). The physiotherapist then informs the patient verbally. The randomisation list was constructed prior to the beginning of the study by an off-site independent statistician with no clinical involvement in the study.

Clinical and psychological evaluations are performed by blinded assessors. As randomisation takes place three months after inclusion, the physiotherapist is unaware of group allocation for the first two evaluations (M0 and M3). Patients are informed not to disclose their allocation to their treating neurologist or psychologist. Throughout the study all participants are requested to maintain their regular lifestyle, the only difference being the three-month exercise period for the experimental group which is lacking for the control group.

### Intervention

**Fig 3:**
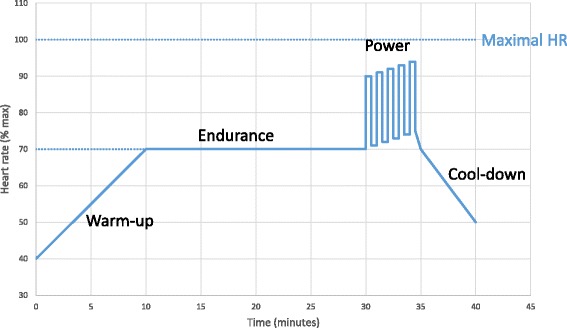
Theoretical profile of a training session

Patients randomised to the experimental group (EX) participate in two to three supervised exercise sessions to learn how to use the rowing machine (Concept2™) and follow the individualised exercise program. The Concept 2 rowing machine is a stationary ergometer where the patient is seated and simultaneously exercises both upper and lower limbs and the trunk thus targeting the multiple muscles which can be affected in MG. The rowing exercise is impact-free so unlikely to cause pain or injury and the basic stroke is easy to learn even for those untrained and unexperienced with the technique. The machine is then delivered to their home where they continue the sessions three times a week for three months (total of 36 planned sessions). Figure 3 shows the theoretical training session profile.

Each 40-min moderate intensity training session consists of a 10-min warm-up to reach their individual target heart rate (i.e. 70% of their maximal heart rate [HR], using 220-age as HRmax), followed by an endurance plateau phase consisting of 20 min of constant aerobic activity rowing at their individual target heart rate, followed by a power interval phase (ten pulls at maximum effort every minute for 5 min) and finally a 5-min active cool-down period whereby the rowing exercise is continued at a slow pace. A heart rate monitor (Garmin®) is worn by the patient and data (rowing distance, session duration, HR) are recorded by the rowing machine for each training session. These will be analysed to evaluate tolerance and possible cardiovascular adaptations from exercising. Patients are advised to organise their training sessions on alternate days. The first home session is supervised when possible. Details will be presented as per current recommendations for reporting of exercise interventions [30]. Patients continue to benefit from usual care.

### Control group

The control group does not participate in any organised physical exercise program. The control group has no added intervention other than usual care and the scheduled monthly appointments as per the study protocol.

### Compliance to the physical exercise program

Compliance is calculated as the number of completed sessions compared with the number of intended sessions. Session duration and rowing distance are recorded for each session onto a card via the rowing machine monitor. In addition, patients are requested to fill in a training logbook to record medication and any minor adverse effects related to training sessions (such as fatigue or any MG symptoms). Patients are informed to contact their general practitioner if there is any worsening of symptoms and to inform the physiotherapist of the study. Compliance is facilitated by weekly phone calls to check on progress and provide encouragement. Reasons for non-compliance are noted. The rowing machine is collected at the end of the intervention period (i.e. M6). Factors preventing or limiting completion of the exercise program are noted such as absence, unrelated illness, etc.

### Criteria for discontinuing

Patients are advised to stop training if there is a clear exacerbation of their MG or if there is any other medical reason or new event for which the exercise program is contraindicated.

### Outcome measures

Safety factors are evaluated monthly and muscular and psychological status is evaluated at baseline and at three, six and nine months (Fig. 2).

## Training effect

### Primary outcome measure

The various potential benefits of exercise training can be appreciated by a change in one’s QOL which incorporates multiple dimensions of a person. A disease-specific QOL measure evaluates the perceived impact of the disease on one’s QOL. The *MGQOL-15-F* is an MG-specific self-administered QOL scale. It evaluates the perceived impact of MG on one’s QOL over the preceding weeks.

The *MGQOL-15-F* was translated from English and the French version has recently been validated in a large cohort of patients [31]. The questionnaire consists of 15 items scored on a 5-point Likert scale (from 0 to 4). Scores are in the range of 0–60 with a higher score indicating a lower QOL. It is both reliable and valid and considered to be an excellent measure of perceived QOL in MG [28]. The primary outcome is the mean change in total *MGQOL-15-F* score at 3–6 months, i.e. before and after the intervention period. The questionnaire is given to and collected from participants by blinded assessors.

### Secondary outcome measures

#### Clinical, biological and pharmacological assessment

MG medical history, including thymectomy, medications, duration of the disease, disease course (exacerbations and crisis) and symptoms, is noted at baseline. The severity and type of MG is classified according to the MGFA classification and the MGFA post-intervention status is used to describe current status of symptoms and treatment [29].

A monthly assessment performed by a blinded neurologist involves a general check-up including vital signs (blood pressure, HR) and cardiopulmonary auscultation, biological markers from venous blood samples and an electrocardiogram. A thorough subjective patient history is carried out including MG behaviour over the preceding month and any other symptoms such as muscle or joint or chest pain or other illness. The frequency and severity of exacerbations are noted, as part of the regular clinical assessment and reported as per the safety criteria of the study. Medication type and dosage is noted and changes are made as per the clinical judgement of the treating neurologist. All neurologists are specialised in managing MG.

Disease severity is evaluated with the Myasthenic Muscle Score (MMS), a performance-based measure of myasthenic symptoms [32, 33]. The score consists of nine items including strength and endurance of limbs, face, eyes and bulbar symptoms. The total maximum attainable score is 100 and a lower score indicates greater disease severity. The mean change in MMS score between three and six months will be evaluated. The impact of symptoms on daily activities is evaluated with the MG-ADL scale [34]. The MG-ADL is a patient-reported scale comprising eight items rated on a 4-point Likert scale evaluating the functional limitations due to myasthenic symptoms over the preceding eight days. The mean change in MG-ADL score between three and six months will be evaluated. Change in cumulated corticosteroid and anticholinesterase inhibitor dosage between three and six months is recorded as a secondary outcome measure.

#### Strength and endurance measures

All strength and endurance measures take place in the same neuromuscular evaluation laboratory (Neuromuscular Physiology and Evaluation Lab, Institute of Myology, Pitié- Salpêtrière Hospital, Paris) by the same trained physiotherapist.

#### Pulmonary function and respiratory strength

For all respiratory evaluations, patients are seated with a backrest and feet firmly on the floor. Forced vital capacity (FVC) is measured with a Micro Medical Spiro USB spirometer and Spida 5 software or a Vitalograph spirometer (the same spirometer is used for all evaluations for each patient). Calibration is performed according to manufacturer’s guidelines with a 3-L syringe before each evaluation. FVC in litres is measured from total lung capacity according to the ATS/ERS task force guidelines [35]. Patients are instructed to inhale completely and then exhale maximally until no more air can be expelled. For respiratory muscle strength evaluations, the MicroRPM device (Micromedical, Rochester, UK) is used. Maximal inspiratory pressure (MIP) is measured first, followed by maximal expiratory pressure (MEP). Both are expressed in cmH_2_O. MIP is measured from residual lung volume and MEP from total lung capacity [36]. All measurements are repeated three times or until maximum values are reached if the patient continues to improve and remains clinically stable (no signs of fatigue). For MEP measures, patients are asked to place their hand around the mouthpiece to minimise air leaks. A tubed mouthpiece is used for both. Strong verbal encouragement is provided for all measures. A nose clip is used for FVC. The highest value from the three trials is used in data analysis. Secondary outcome measurements involve the mean change in FVC, MIP and MEP between M3 and M6.

#### Upper and lower limb strength and fatigue

Voluntary strength testing is performed using a Biodex System 3 Pro dynamometer (Biodex Medical Systems, Shirley, NY, USA). For the upper limb, elbow flexion force is evaluated. The patient is seated in the adjustable Biodex chair with the shoulder in 90° flexion and 15° horizontal abduction. The upper arm is placed on a support, the elbow flexed to 90° and the forearm in full supination, with the strap placed at the wrist, leaving the hand free. The axis of the dynamometer is aligned with the joint axis of rotation of the elbow. Bilateral shoulder straps and a waist strap are used to stabilise the thorax and pelvis to avoid compensations. For the lower limb knee extension force is evaluated with the patient seated in the Biodex chair with the hips and knees flexed to 90°. The thorax, pelvis and right thigh are secured with straps. The axis of the dynamometer is aligned with the axis of rotation of the knee. Both positions are designed to avoid the influence of gravity on the muscle contraction.

Before each test, there is one test trial at submaximal force to ensure that the patient understands the instructions. Testing involves three isometric contractions at maximal voluntary isometric force (MVIC) followed by a 1-min rest between each MVIC trial. Maximal torque is presented in Nm. The highest value from the three trials of each muscle group is used in data analysis. Following the third MVIC there is a 5-min rest period where the patient remains in the same position. Fatigue testing comprises a sustained 40-s isometric contraction at 50% of their best individual MVIC from the same day. A last MVIC is performed immediately after completing the fatigue exercise. Visual cues are provided to the patient via a computer screen to enable them to maintain the target level of force for the duration of the fatigue test. Strong verbal encouragement is provided by the evaluator for both force and fatigue testing. The details of the dynamometer position (chair position, height, depth, etc.) for each patient are noted for repeatability in successive testing. Measurements are performed on the right side only. Secondary outcome measure is the mean change in UL and LL strength between M3 and M6.

#### Electrophysiological measurements

Surface EMG (sEMG) is a non-invasive method of evaluating the electrical signals of muscles to gain insight into the physiological characteristics of a muscle contraction. It has been used to investigate peripheral muscle fatigue in various conditions [37, 38] Myoelectrical manifestations of fatigue can be studied during sustained submaximal contractions through changes in amplitude and frequency content of the sEMG signal. In this study, four aligned miniature, circular silver-silver chloride (Ag/AgCl) surface electrodes of 4 mm in diameter, 11 mm inter-electrode distance are used. Three signals are derived from this bipolar configuration. The electrodes are connected to an amplifier (bandwidth = 10–1000 Hz). Where necessary, hair is gently removed with a shaver and the skin is abraded slightly and cleaned with an alcohol solution to reduce inter-electrode resistance. To improve conduction electrolytic gel is placed on the electrodes which are then placed on the biceps brachii (BB) for elbow flexion and on the vastus lateralis (VL) for knee extension, parallel to the estimated direction of muscles fibres according to the Surface EMG for Non-Invasive Assessment of Muscles (SENIAM) recommendations [39]. A neutral reference electrode is placed on the ulnar styloid for the BB and the fibular head or patella for the VL.

Surface EMG recordings are performed for both force and fatigue tests at a sampling rate of 10 kHz and stored on the computer for further analysis. Surface EMG recordings will be processed using standard methods using the root mean square (RMS) as an estimate of the signal amplitude, notably indicating the level of motor unit recruitment during the contraction and the mean power frequency (MPF) as an estimate of the mean value of the power spectral distribution of the signal used to evaluate localised muscle fatigue. The onset of fatigue is identified by a decrease in the MPF. Where possible, muscle fibre conduction velocity will also be computed.

#### Grip strength

Handgrip strength of the dominant hand is measured via an isometric contraction using the Myogrip dynamometer (Ateliers Laumonier, France), an electronic device specifically designed to measure grip strength in neuromuscular disorders [40]. Its handle can be adjusted to accommodate different hand sizes and the strength is displayed directly on the screen. Patients are seated on the edge of a plinth, their back in an upright position, hips and knees flexed to 90° and feet flat on the floor. The dominant arm hangs down the side of the body with the shoulder in neutral and the elbow extended. The patient is instructed to squeeze the handle as hard as possible; verbal encouragement is provided to motivate the patient to perform a maximal effort. The test is performed three times or until no further improvement. Maximal grip strength is recorded in kg. Mean change in grip strength between M3 and M6 is a secondary outcome.

#### Walking endurance

Walking endurance is measured via the 6-min walk test (6MWT), a simple, well tolerated and reliable quantitative measure of functional exercise capacity [41]. Patients are instructed to walk the longest distance possible in 6 min in a 25 m long, level surface indoor corridor. At each end of the 25 m they turn around a cone placed on the floor. There is a starting line marked on the floor on the right of the first cone. Instructions provided before the test and verbal guidance throughout the test are standardised according to the American Thoracic Society guidelines/recommendations [42]. The total distance (m) is recorded (6MWD) as well as the time every 25 m. The patient is advised to wear comfortable walking shoes for the test. An accelerometer (Locometrix) is worn around the lumbar spine during the test for possible more in-depth gait analyses. Mean change in 6MWD between M3 and M6 will be evaluated as a secondary outcome.

As anticholinesterase inhibitors can temporarily influence muscle strength and fatigue the timing of medication is recorded as well as the timing of each evaluation. Where possible the same timing is respected for consecutive evaluations.

#### Free-living physical activity

Free-living physical activity and sedentary behaviour are quantitatively measured at four time points during the trial. Patients wear a tri-axial seismic accelerometer (DynaPort Movemonitor, McRoberts) on their lumber spine using the supplied elastic belt. The accelerometer weighs 55 g and measures 106.6 × 58 × 11.5 mm. It stores raw data on a microSD card and contains a USB connection to upload data to the software for preparation and analysis. The accelerometer measures acceleration in three axes at a sample rate of 100 samples per second. Algorithms developed by the manufacturer quantify physical activity and inactivity by measuring body posture and movement intensity from acceleration signals [43]. Body postures include lying/sitting/locomotion/standing and shuffling where shuffling is defined as < 3 consecutive steps or where the intensity or direction of motion doesn't comply with locomotion. Measurements begin at a pre-set time. Patients are instructed to wear the device during waking hours for seven consecutive days, except when bathing/showering or swimming. Multiple days are monitored to account for day-to-day variation.

Free-living physical activity is further evaluated via the completion of a Bouchard questionnaire at the beginning of the study [44]. The questionnaire enables an estimation of the daily energy expenditure over a three-day period by asking patients to record their physical activity over a 72-h period (two weekdays and one weekend day). The day is divided into 96 periods of 15 min and activities are quantified on a scale of 1–9 depending on their intensity. The questionnaire has the added benefit of providing a qualitative perspective on the activities performed which is lacking in the accelerometer data. Yet the accelerometer data, when worn correctly, lacks the problems of recall bias. Thus, the two can be considered complementary.

#### QOL and psychological state

At three, six and nine months anxiety is evaluated via the State Trait Anxiety Inventory (STAI) and depression is evaluated via the modified Beck Depression Inventory (BDI-13). Psychiatric disorders are evaluated by the Mini-International Neuropsychiatric Interview (MINI, French Version 5.0.0) and self-esteem via the Self-Esteem Inventory scale (SEI) at both three and nine months. Global QOL is measured via the World Health Organisation QOL-BREF scale and specific QOL via the MGQOL-15-F at zero, three, six and nine months. Semi-structured interviews are conducted at zero, three, six and nine months. We ask patients to draw themselves at zero, six and nine months. Evaluations are carried out during a face-to-face meeting with a clinical psychologist which lasts approximately 90 min. The mean change in the total score for each questionnaire will be compared between groups at M3 and M6 as secondary outcomes.

The STAI evaluates anxiety via a self-administered questionnaire consisting of two 20-item sub-scales: the STAI A evaluates the actual level of anxiety at the moment of the assessment (state anxiety); and the STAI B investigates more general and long-standing anxiety (trait anxiety) [45]. Scores are in the range of 20–80 for each questionnaire with higher scores indicating greater anxiety. The French version was validated by Bruchon-Schweitzer and Paulhan [46].

The SEI evaluates the judgment that an individual holds about themselves regardless of the circumstances. The self-administered questionnaire, adapted into French (ECPA, 1984), consists of 58 items with sentences describing feelings, opinions or individual reactions with either a ‘like me’ or ‘unlike me’ response [47]. It investigates self-esteem in four domains: personal (26 items); social (eight items); family (eight items); professional (eight items); and contains a lie scale (eight items). A higher score indicates greater self-esteem.

Depression is evaluated via the self-administered BDI-13 (short version), consisting of 13 items validated in French by Collet and Cottraux [48]. Each item has four response choices (0–3) and provides a global score (0–39) corresponding to depression severity (0–4 = no depression, 4–7 = mild, 8–15 = moderate, ≥ 16 = severe depression).

The MINI is a short, structured, clinical interview organised in diagnostic modules [49]. It enables researchers to investigate 16 axis I DSM-IV disorders according to the DSM-IV or the CIM-10. For most modules, two to four screening questions are used to rule out the diagnosis when answered negatively. Positive responses to screening questions are explored by further investigation of other diagnostic criteria. The MINI is also used to confirm the diagnosis of depression if the Beck score is ≥ 4.

Global QOL is evaluated by the WHOQOL-BREF [50]. The French translation was validated by Leplège et al. in patients with various neuromuscular diseases [51]. This self-administered questionnaire consists of 26 items (score of 0–5) and assesses QOL according to four domains: physical health; psychological health; social relationships; and environment. Myasthenia-specific QOL is evaluated by the French version of the Myasthenia Gravis QOL scale, the MGQOL-15-F as previously described (primary outcome measure) [31].

Semi-structured interviews are conducted by trained clinical psychologists to qualitatively assess the psychological impact of MG on patients’ personal, marital, social, professional life and physical activities. The interviews are recorded to be qualitatively analysed with the NVivo software 7 as it allows a thematic analysis [52]. Finally, patients are asked to draw themselves. The instructions are: ‘Can you draw yourself as you feel today?’ This drawing is analysed as a projective test and considered as an index of the representation of the body and of its investment [53].

### Other exploratory measures

#### Inflammatory and immunological markers

The level of pro- and anti-inflammatory cytokines is measured at three, six and nine months. A total of 50 mL of venous blood is collected and the serum and mononucleated cells are isolated. Blood samples of the same patients are operated simultaneously to avoid inter-experimental variability. The sera are conserved in a freezer at – 80 °C and the cytokines are analysed by ELISA or Bioplex technology. The cytokines to be studied in the sera include the interleukin (IL)-6, IL-17, IL-21, interferon gamma and tumour necrosis factor alpha (pro-inflammatory cytokines) as well as IL-10 and IL-1RA (anti-inflammatory cytokines) [54]. The mononucleated cells are isolated using a Ficoll gradient. The cells are frozen in a freezing medium containing 10% dimethyl sulfoxide. The phenotype of the monocytes and lymphocytes will be investigated by flow cytometry using a combination of eight markers. The expression of cluster of differentiation (CD)14 coupled with CD16 and Toll-like receptor 4 of the monocytes will be analysed [54]. We will also determine the proportion of regulatory B cells (Breg) and regulatory T cells (Treg) in the lymphocytes. The percentage of Treg cells will be determined using the combined expression of CD4, CD25, CD127 and FoxP3 (a marker associated with the regulatory function) on permeabilised cells (as the FoxP3 marker is intracellular). The percentage of Breg cells will be determined by the combination of CD19, CD38, CD27 and CD24. The expression of chemokine receptors such as CXCR3 (pro-inflammatory) and CXCR5 (follicular helper cells) will also be determined. The flow cytometry experiments will be performed on a FACSCanto (Becton-Dickinson) apparatus.

Levels of circulating specific MG antibodies (AChR or MuSK) are measured at inclusion, three, six and nine months.

### Adverse event reporting

An adverse event (AE) is defined as any untoward medical occurrence in a participant which does not necessarily have a causal relationship with this study. These can be identified at any moment during the study. Eligibility criteria rule out patients with known contraindications to physical exercise. An ECG is performed monthly to monitor cardiac status. It is expected that patients in the experimental group may experience muscle soreness and fatigue in response to exercise, particularly for the more sedentary patients. However, this is not an AE as it is considered a normal response to exercise.

A serious adverse event (SAE) includes: death; risk of death; necessity or prolongation of existing hospitalisation; persistent or significant disability or incapacity; or any other undesirable event considered to be medically significant. MG exacerbation requiring modification of current therapeutic management during the intervention period is considered a possible AE. All suspected unexpected SAEs are to be notified without delay to the sponsor, as well as any worsening of the disease suspected to be related to the physical programme if a patients' treatment needs to be modified. In this case, unblinding can occur. Should a worsening occur without needing a change of the treatment, it is only to be recorded in the electronic database without mandating a direct report to the sponsor. The sponsor’s vigilance unit is in charge of the causality assessment as well as of its regulatory duties with regard to reporting obligations to the French Medicines Agency.

### Data and statistical analysis

#### Data management

Data are recorded and managed through a dedicated web-based software (Cleanweb Telemedicine) with secured and restricted access. Access to the system is controlled for each investigator by an individual login/password and using a secured https connection. Data are stored on a centralised server. Participants also have paper files which are anonymised and only contain the participant’s unique identification code. These are stored in a dedicated storage unit in each centre. Access to the complete final trial dataset will be restricted to the Clinical Research Unit statistician who will analyse the study data for the purpose of report and publication. Subsets of the final dataset may be shared with investigators if needed for discussion or additional analyses. Should the need arise, advice from the steering Committee will be sought and provided to the sponsor APHP who will retain ownership of the data as well as the final decision on authorising further access to data.

### Monitoring

Clinical research assistants from the sponsors research unit regularly visit all centres for on-site monitoring to ensure protocol compliance and monitor data quality according to the data management unit guidelines and clarification forms. Data entered into Cleanweb should be complete and consistent with source documents. Eligibility criteria and consent forms are checked as well as outcome measures and adverse events. No interim analyses are planned.

### Statistical analysis

The statistical analysis will be performed once the planned number of patients will have completed the study, according to the intention-to-treat principle. Each participant is analysed according to the group in which he/she was randomised. For the primary outcome (*MGQOL-15-F* at three or six months*)*, missing values will be handled through multiple imputation by chained equations. No multiple imputation will be carried out for secondary outcomes. The main analysis of the primary and secondary outcome measures will not be adjusted. Adjusted analyses on the centre will be performed but considered as secondary analyses. As there are only two centres, the centre will be modelled as a fixed effect. Two-sided tests will be used with a 5% significance level.

#### Patient description and follow-up

A descriptive analysis of inclusions and follow-up will be performed (graph of inclusions and theoretical and actual visits). All patients will be included in the analysis unless their consent is retracted and they are opposed to their data being analysed. Any drop-outs or patients having stopped the intervention early will be included in the analysis. A patient is considered a drop-out when he/she revokes his/her consent to participate in the study and thus all participation in the study will cease.

Epidemiological and clinical characteristics of patients at baseline will be described by group without statistical tests. Any violation to the protocol and reasons for drop out will be described.

The primary outcome measure, the changes in *MGQOL-15-F*, will be analysed via a Student’s test and adjustment on the centre will use ANOVA. If the distribution of the changes in the *MGQOL-15-F* between the two groups is very asymmetrical, then a non-parametric approach based on the ranks will be used for statistical testing.

#### Secondary outcome measure analysis

For binary outcome measures a non-adjusted analysis will be carried out using Fisher’s exact tests. The analysis will then be adjusted according to the centre via a logistic regression model. The quantifiable outcome measures will be analysed using a Student’s test for the non-adjusted analysis and an ANOVA model for the analysis adjusted for the centre. A transformation (for example a logarithm) will eventually be envisaged if the distribution of variables is not compatible with the normal distribution assumption. If the envisaged transformations do not enable a symmetrical distribution, a non-parametric analysis will be used (Wilcoxon test and ANOVA on ranks).

The analysis will be performed using the R software (version 3.4.3 or later; The R foundation for statistical computing, Vienna, Austria).

### Timeline

Recruitment and final inclusions are anticipated to be completed in June 2017 with final subjects completing the study in February 2018.

### Dissemination

We will provide all participants with a summary of trial outcome once data analysis has been completed. The trial results will be widely disseminated to the scientific community and the general community via publications in international peer-reviewed journals and presentations in national and international conferences as well as disseminated to patients via patient groups.

## Discussion

In this article, we describe the design and methods of MGEX, a multi-centre, two-arm, parallel group, randomised, controlled trial investigating the tolerance and potential benefits of a moderate-intensity, individualised, home-based physical exercise program in adults with generalised, stable, MG. This is the first study to explore physical exercise in this population with such a large sample size and rigorous methodological design.

Another strength of this study is the comprehensive evaluation using robust outcome measures. Outcome measures are valid and reliable and explore the multiple facets of the disease including the psychological aspects, the various physical symptoms, from a clinical, functional and physiological perspective as well as the complex immunological aspects.

Eligibility criterion are broad enabling the inclusion of multiple MG phenotypes which is important for the external validity of results, particularly as MG is a heterogeneous disease.

Recommendations regarding the practice of physical exercise in this population are lacking. Both patients and clinicians are awaiting evidence-based guidelines. The results of this study have the potential to provide much awaited and needed insight into the feasibility, tolerability and potential beneficial effects of physical exercise for patients with stabilised MG. Another non-randomised trial evaluating multi-modal exercise in stable MG is underway (NCT01047761) and due for completion in 2020.

If safe, participating in regular physical exercise may not only contribute to minimising deconditioning but may also improve QOL and daily function for patients and contribute to secondary prevention of various chronic conditions such as cardiovascular disease, cancer and depression. Moreover, the possible immunomodulatory effects of exercise which have been investigated in other auto-immune diseases, may also play an important role in the management of this auto-immune disease.

As quantitative data will be collected from the physical exercise sessions in MGEX, there will be no recall bias problems in relation to adherence and tolerability evaluations. The exercise program is personalised and is carried out at home, unsupervised, which is also important for real-life transfer. Thus, patients can be more self-sufficient and autonomous in undertaking an active lifestyle.

### Trial status

To date, 32 patients have been included. Recruitment began in October 2014 and remains open and we are optimistic that the required number of participants will be met. No AEs related to exercise have been reported at this stage. This is V3.0 of the protocol, last updated on 12 January 2017. Clinical research assistants are responsible for informing all necessary bodies of any protocol modifications.

## Additional file

Additional file 1: SPIRIT checklist. (PDF 280 kb)

## Abbreviations

ANOVA: Analysis of variance
CD: Cluster of differentiation
ECG: Electrocardiogram
FVC: Forced vital capacity
HR: Heart rate
IL: Interleukin
MEP: Maximal expiratory pressure
MG: Auto-immune myasthenia gravis
MG-ADL: MG activities of daily living
MG-QOL-15: MG quality of life
MIP: Maximal inspiratory pressure
MMS: Myasthenic muscle score
QOL: Quality of life
WHO-QOL BREF: World Health Organization Quality of Life 26-item questionnaire.
