# Supplementary material for: The benefits and tolerance of exercise in myasthenia gravis (MGEX): study protocol for a randomised controlled trial
Source: Trials. 2018 Jan 18;19:49. doi: 10.1186/s13063-017-2433-2 (PMC5774148; doi:10.1186/s13063-017-2433-2)
Supplement: Additional file 1: — SPIRIT checklist. (PDF 280 kb) [file 13063_2017_2433_MOESM1_ESM.pdf]

SPIRIT 2013 Checklist: Recommended items to address in a clinical trial protocol and related documents*

Section/item	Item No	Description	Addressed on page number
**Administrative information**			
Title	1	Descriptive title identifying the study design, population, interventions, and, if applicable, trial acronym	_page 1_______
Trial registration	2a	Trial identifier and registry name. If not yet registered, name of intended registry	page 4__
	2b	All items from the World Health Organization Trial Registration Data Set	_p7 of checklist/additional file
Protocol version	3	Date and version identifier	p25 of manuscript, details p8 checklist
Funding	4	Sources and types of financial, material, and other support	__page 26
Roles and	5a	Names, affiliations, and roles of protocol contributors	__page 1, 26-7

responsibilities	5b	Name and contact information for the trial sponsor	Mrs. Florence Favrel-Feuillade, Head of the APHP Clinical Research And Innovation Direction florence.favrel-feuillade@drc.aphp.fr
	5c	Role of study sponsor and funders, if any, in study design; collection, management, analysis, and interpretation of data; writing of the report; and the decision to submit the report for publication, including whether they will have ultimate authority over any of these activities	_____p26__
	5d	Composition, roles, and responsibilities of the coordinating centre, steering committee, endpoint adjudication committee, data management team, and other individuals or groups overseeing the trial, if applicable (see Item 21a for data monitoring committee)	_____________
**Introduction**			
Background and rationale	6a	Description of research question and justification for undertaking the trial, including summary of relevant studies (published and unpublished) examining benefits and harms for each intervention	_page 5-6__
	6b	Explanation for choice of comparators	_page 6___
Objectives	7	Specific objectives or hypotheses	_page_6_____
Trial design	8	Description of trial design including type of trial (eg, parallel group, crossover, factorial, single group), allocation ratio, and framework (eg, superiority, equivalence, noninferiority, exploratory)	___page_7-9__
**Methods: Participants, interventions, and outcomes**			

Study setting	9	Description of study settings (eg, community clinic, academic hospital) and list of countries where data will be collected. Reference to where list of study sites can be obtained	___page_8_____
Eligibility criteria	10	Inclusion and exclusion criteria for participants. If applicable, eligibility criteria for study centres and individuals who will perform the interventions (eg, surgeons, psychotherapists)	__page_8-9__
Interventions	11a	Interventions for each group with sufficient detail to allow replication, including how and when they will be administered	__page_10-11
	11b	Criteria for discontinuing or modifying allocated interventions for a given trial participant (eg, drug dose change in response to harms, participant request, or improving/worsening disease)	_page_11___
	11c	Strategies to improve adherence to intervention protocols, and any procedures for monitoring adherence (eg, drug tablet return, laboratory tests)	___page_11_
	11d	Relevant concomitant care and interventions that are permitted or prohibited during the trial	____
Outcomes	12	Primary, secondary, and other outcomes, including the specific measurement variable (eg, systolic blood pressure), analysis metric (eg, change from baseline, final value, time to event), method of aggregation (eg, median, proportion), and time point for each outcome. Explanation of the clinical relevance of chosen efficacy and harm outcomes is strongly recommended	__page_11-21_
Participant timeline	13	Time schedule of enrolment, interventions (including any run-ins and washouts), assessments, and visits for participants. A schematic diagram is highly recommended (see Figure)	__figure 1 -2
Sample size	14	Estimated number of participants needed to achieve study objectives and how it was determined, including clinical and statistical assumptions supporting any sample size calculations	___page_7___
Recruitment	15	Strategies for achieving adequate participant enrolment to reach target sample size	__page 8__

Methods: Assignment of interventions (for controlled trials)

Allocation:

Sequence generation	16a	Method of generating the allocation sequence (eg, computer-generated random numbers), and list of any factors for stratification. To reduce predictability of a random sequence, details of any planned restriction (eg, blocking) should be provided in a separate document that is unavailable to those who enrol participants or assign interventions	___page_9-10__

Allocation concealment mechanism	16b	Mechanism of implementing the allocation sequence (eg, central telephone; sequentially numbered, opaque, sealed envelopes), describing any steps to conceal the sequence until interventions are assigned	page_9-10__
Implementation	16c	Who will generate the allocation sequence, who will enrol participants, and who will assign participants to interventions	__page_9-10___
Blinding (masking)	17a	Who will be blinded after assignment to interventions (eg, trial participants, care providers, outcome assessors, data analysts), and how	__page_9-10__
	17b	If blinded, circumstances under which unblinding is permissible, and procedure for revealing a participant's allocated intervention during the trial	Page 21

Methods: Data collection, management, and analysis

Data collection methods	18a	Plans for assessment and collection of outcome, baseline, and other trial data, including any related processes to promote data quality (eg, duplicate measurements, training of assessors) and a description of study instruments (eg, questionnaires, laboratory tests) along with their reliability and validity, if known. Reference to where data collection forms can be found, if not in the protocol	__page 11-21
	18b	Plans to promote participant retention and complete follow-up, including list of any outcome data to be collected for participants who discontinue or deviate from intervention protocols	____N/A_______
Data management	19	Plans for data entry, coding, security, and storage, including any related processes to promote data quality (eg, double data entry; range checks for data values). Reference to where details of data management procedures can be found, if not in the protocol	_page_21-22____
Statistical methods	20a	Statistical methods for analysing primary and secondary outcomes. Reference to where other details of the statistical analysis plan can be found, if not in the protocol	page_22-4__
	20b	Methods for any additional analyses (eg, subgroup and adjusted analyses)	_page 23___
	20c	Definition of analysis population relating to protocol non-adherence (eg, as randomised analysis), and any statistical methods to handle missing data (eg, multiple imputation)	__page_23__

Methods: Monitoring

Data monitoring	21a	Composition of data monitoring committee (DMC); summary of its role and reporting structure; statement of whether it is independent from the sponsor and competing interests; and reference to where further details about its charter can be found, if not in the protocol. Alternatively, an explanation of why a DMC is not needed	______
	21b	Description of any interim analyses and stopping guidelines, including who will have access to these interim results and make the final decision to terminate the trial	______
Harms	22	Plans for collecting, assessing, reporting, and managing solicited and spontaneously reported adverse events and other unintended effects of trial interventions or trial conduct	_______
Auditing	23	Frequency and procedures for auditing trial conduct, if any, and whether the process will be independent from investigators and the sponsor	_____N/A______
**Ethics and dissemination**			
Research ethics approval	24	Plans for seeking research ethics committee/institutional review board (REC/IRB) approval	page_26___
Protocol amendments	25	Plans for communicating important protocol modifications (eg, changes to eligibility criteria, outcomes, analyses) to relevant parties (eg, investigators, REC/IRBs, trial participants, trial registries, journals, regulators)	_p25______
Consent or assent	26a	Who will obtain informed consent or assent from potential trial participants or authorised surrogates, and how (see Item 32)	____
	26b	Additional consent provisions for collection and use of participant data and biological specimens in ancillary studies, if applicable	_________
Confidentiality	27	How personal information about potential and enrolled participants will be collected, shared, and maintained in order to protect confidentiality before, during, and after the trial	__page 22___
Declaration of interests	28	Financial and other competing interests for principal investigators for the overall trial and each study site	__page 26____
Access to data	29	Statement of who will have access to the final trial dataset, and disclosure of contractual agreements that limit such access for investigators	__ page 22 _______

Ancillary and post-trial care	30	Provisions, if any, for ancillary and post-trial care, and for compensation to those who suffer harm from trial participation	____ ______
Dissemination policy	31a	Plans for investigators and sponsor to communicate trial results to participants, healthcare professionals, the public, and other relevant groups (eg, via publication, reporting in results databases, or other data sharing arrangements), including any publication restrictions	____page_24___
	31b	Authorship eligibility guidelines and any intended use of professional writers	___________
	31c	Plans, if any, for granting public access to the full protocol, participant-level dataset, and statistical code	___ ________
**Appendices**			
Informed consent materials	32	Model consent form and other related documentation given to participants and authorised surrogates	___________
Biological specimens	33	Plans for collection, laboratory evaluation, and storage of biological specimens for genetic or molecular analysis in the current trial and for future use in ancillary studies, if applicable	_____

*It is strongly recommended that this checklist be read in conjunction with the SPIRIT 2013 Explanation & Elaboration for important clarification on the items. Amendments to the protocol should be tracked and dated. The SPIRIT checklist is copyrighted by the SPIRIT Group under the Creative Commons "Attribution-NonCommercial-NoDerivs 3.0 Unported" license.

Item 2b WHO Data Set

DATA CATEGORY	INFORMATION[32]
Primary registry and trial identifying number	ClinicalTrials.gov<br>NCT02066519
Date of registration in primary registry	January 13, 2014
Source(s) of monetary or material support	French Ministry of Health via the *Regional Programme for Hospital Clinical Research* grant with collaboration from the Association Institute of Myology
Primary sponsor	APHP
Contact for public queries	Tarek SHARSHAR
Contact for scientific queries	Tarek SHARSHAR
Scientific title	The benefits and tolerance of exercise in Myasthenia Gravis: MGEX, a study protocol for a randomised, controlled trial.
Countries of recruitment	France
Health condition(s) or problem(s) studied	Acquired Myasthenia Gravis
Intervention(s)	Active comparator: physical exercise program Placebo comparator: usual care

Item 3 Protocol Version Information

This manuscript describes V2 of the protocol.

	**ANSM**			**CPP**			**Protocol**			**NIFC (Information and consentment notice)**		
	Reference	Submission	Response	Référence	Submission	Response	version	date	application	version	date	application
**Initial request**	131300B-32	02/08/2013	11/10/2013	13064	25/09/2013	12/12/2013	**V1.1**	05/11/2013	12/12/2013	**V1.0**	12/09/2013	12/12/2013
**Modification 1**	131300S-3201 MS1	15/01/2015	08/02/2015	13064/MS1	02/02/2015	13/03/2015	**V2.0**	15/01/2015	13/03/2015	**V2**	15/01/2015	13/03/2015
**M2**				13064/MS2	21/06/2016	26/09/2016	**V2.0**	15/01/2015	26/09/2016	**V2**	15/01/2015	26/09/2016
**M3**				13064/MS3	12/01/2017	13/03/2017	**V3.0**	12/01/2017	13/03/2017			

	**Important Modifications**	**Inclusion criteria**	**Non inclusion criteria**	**Inclusion period**	**Others**
**M1**	• Suppression of criteria : stabilisation by prédnisone and/or azathioprine<br>• Modification of the clinical description of myashtenia symptoms, decrement > 10% on EMG and positive prostigmine test<br>• Age max modification (change from 60 to 70 years old)<br>• Suppression of non inclusion criteria : physical exercise > 1h30<br>• Modification systolic ejection fraction < 50%<br>• Modification quality of life score MGQOL-15 30 to 15/60<br>• Modification number of patients from 44 to 42<br>• Modification time point cytokine dosage<br>• Modification of the organization of physical activity	X	X		X
**M2**	• Neurologie 1 service added (Pitie Salpetriere)				X
**M3**	• Inclusion period extended 12 months:<br>- Total duration of inclusions changed from 27 to 39 months<br>- Total duration of the research changed from 36 to 48 months			X	
